# Novel lipid profiles and atherosclerotic cardiovascular disease risk: insights from a latent profile analysis

**DOI:** 10.1186/s12944-025-02471-3

**Published:** 2025-02-25

**Authors:** Hongli Wan, Haisheng Wu, Yuxi Wei, Simin Wang, Yuqiang Ji

**Affiliations:** 1 Department of Central Laboratory, Xi’an No.1 Hospital, The First Affiliated Hospital of Northwest University, Xi’an City South Street powder Lane No. 30, Xi’an, Shaanxi 710002 China; 2 Xi’an Key Laboratory for Innovation and Translation of Neuroimmunological Diseases, Xi’an No.1 Hospital, Xi’an City South Street powder Lane No. 30, Xi’an, Shaanxi 710002 China; 3 School of Public Health, LKS Faculty of Medicine, The University of Hong Kong, Hong Kong Special Administrative Region, 999077 China

**Keywords:** Lipids, Atherosclerosis, Coronary artery disease, Latent class analysis

## Abstract

**Background:**

Dyslipidemia is a key contributor to atherosclerotic cardiovascular disease (ASCVD). Despite the well-established correlation between abnormal lipid metabolism and ASCVD, existing diagnostic and predictive models based on lipid indices alone or in combination often exhibit suboptimal sensitivity and specificity. There is an urgent need for improved lipid indicators or novel combinations thereof.

**Methods:**

The study included 898 cardiology inpatients who underwent coronary angiography (CAG). A latent profile analysis (LPA) was utilized to delineate lipid profiles on the basis of four routine lipid indices (total cholesterol (TC), low-density lipoprotein (LDL), high-density lipoprotein (HDL), and triglycerides (TG)) and the triglyceride‒glucose (TyG) index as a proxy for the TG. Logistic regression models were used to assess the correlations between lipid profiles and the occurrence and severity of coronary artery stenosis (CAS and severe CAS), as well as the occurrence of coronary heart disease (CHD). Predictive modeling subsequently validated the predictive power of the lipid profiles for cardiovascular outcomes.

**Results:**

The LPA delineated four distinct lipid profiles: *Profile 1* (relatively high HDL with the lowest TC, LDL and TyG, 41.20%), *Profile 2* (relatively high TC, LDL, and TyG with the lowest HDL, 36.42%), *Profile 3* (relatively low TC, LDL and TyG with relatively high HDL, 18.93%), and *Profile 4* (the highest TC, LDL, and TyG with the highest HDL, 3.45%). *Profile 1* was associated with the lowest ASCVD risk, whereas *Profile 2* posed the highest risk for all adverse outcomes. The risk associated with *Profile 3* and *Profile 4* varied depending on the outcome. *Profile 4* presented a lower odds ratio (OR) for CAS than did *Profile 3*, whereas *Profile 3* presented a lower OR for severe CAS and CHD. The lipid profile variable substantially outperformed individual lipid indices or their combinations in predicting cardiovascular outcomes.

**Conclusions:**

Four distinct lipid profiles were identified among patients, with a particular profile characterized by lower levels of TC, LDL, and TyG, as well as a lower HDL, emerging as the most predictive of adverse cardiovascular outcomes. This underscores the critical need for a thorough lipid profile analysis to pinpoint individuals at heightened risk for adverse cardiovascular outcomes.

**Supplementary Information:**

The online version contains supplementary material available at 10.1186/s12944-025-02471-3.

## Introduction

Atherosclerotic cardiovascular disease (ASCVD) encompasses a range of cardiovascular conditions and stands as the primary cause of mortality globally [1–2]. Driven by atherosclerosis and inflammation, ASCVD can present in various forms, such as coronary artery stenosis, stable and unstable angina pectoris, sudden cardiac death and myocardial infarction (MI) [3–4]. The pathogenesis of ASCVD is influenced by a synergistic effect of multiple risk factors, with dyslipidemia being the most significant [5]. Dyslipidemia contributes to almost half (49.2%) of the attributable risk for MI, making it a major risk factor for ASCVD [6–8]. Notably, 56.9% of individuals exhibit dyslipidemia [9]. Epidemiological data from China indicate a persistent elevation in dyslipidemia within the adult population, with a nationwide cross-sectional study in 2018 demonstrating that over one-third (35.6%) of Chinese citizens aged 18 years and older were affected [10–11]. Early recognition of dyslipidemia is critical for the timely prevention and diagnosis of ASCVD.

Elevated total cholesterol (TC) levels represent the predominant manifestation among lipid metabolism disorders [12]. Additionally, clinical investigations consistently reveal characteristic lipid profile abnormalities in ASCVD patients, including increased concentrations of triglyceride (TG) and low-density lipoprotein (LDL), coupled with reduced high-density lipoprotein (HDL) concentrations [13–15]. These four lipid indices are routinely tested in blood lipid panels [16–17].

Despite the well-established link between elevated lipid levels and cardiovascular disease, many diagnostic and predictive models relying on individual lipid index or their combination have low sensitivity and specificity. Approximately half of patients with ASCVD with any typical risk factors remain undiagnosed [18], highlighting a significant gap in current diagnostic approaches. Consequently, there is a growing call among researchers for improved lipid index or combinations of indices, which has emerged as a major focus of interest.

Latent profile analysis (LPA) is a sophisticated statistical technique that employs categorical latent variables to uncover hidden subgroups within a population based on a defined set of characteristics [19]. This approach has gained significant attention across various disciplines [20]. It has been utilized to categorize exposure to chemical compounds during pregnancy and to explore the relationships between exposure levels and neurodevelopment in children [21]. In contrast to conventional clustering techniques like *k*-means or hierarchical clustering, which rely solely on observable data, LPA treats an individual’s subgroup membership as a latent categorical variable. This approach assigns individuals to different profiles probabilistically, indicating the degree to which they fit into a particular subgroup. A notable strength of LPA is its capacity to segment populations based on multiple variables, making it highly effective for characterizing complex patterns such as lipid profiles in diverse populations.

In this study, a LPA model was innovatively applied to identify distinct lipid profiles on the basis of four routine lipid indices (TC, LDL, HDL, and TyG, which serve as a proxy for TG). Considerable evidence has demonstrated that the TyG index can effectively predict the risk of various cardiovascular diseases. For instance, it has a significant connection with the onset and development of conditions like CHD, atherosclerosis, atrial fibrillation, heart failure, and stroke [22–24]. Therefore, the TyG index was used as a proxy for TG. By integrating these lipid indices with the Gensini score, which is strongly correlated with the severity of coronary artery disease, this study aimed to provide an outcome-oriented approach that would allow the resulting lipid profile to better reflect clinical outcomes.

By identifying different lipid profiles and examining their associations with coronary artery stenosis (CAS), stenosis severity (severe CAS), and coronary heart disease (CHD), this study aimed to elucidate dyslipidemia classification, as well as its relationship with ASCVD and its role in the diagnosis of ASCVD. The findings of this study could enhance the incorporation of multiple lipid indices into clinical practice, moving beyond the current emphasis on a single lipid index in existing research. This advancement may facilitate the development of more accurate and effective diagnostic and predictive models for ASCVD, potentially leading to better patient care and improved cardiovascular health outcomes.

## Methods

### Study design and populations

**Fig. 1:**
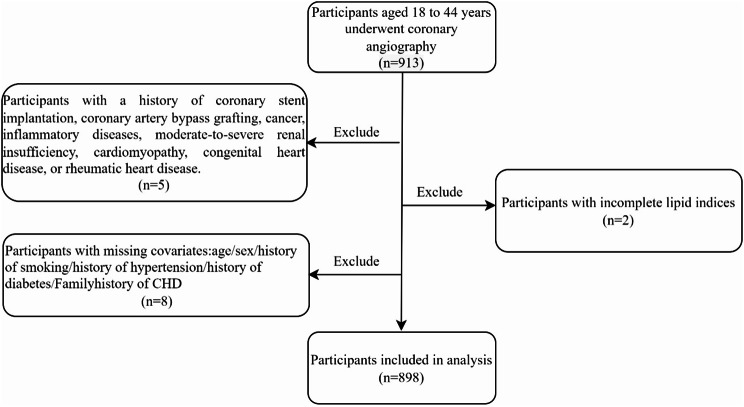
Flowchart of study population

This study retrospectively recruited patients who were hospitalized in the cardiology department of Xi’an No.1 Hospital on account of chest tightness or pain and subsequently underwent coronary angiography (CAG) between January 2020 and March 2024. The indications for coronary angiography included changes in electrocardiograms, myocardial enzyme spectra, and clinical manifestations. All coronary angiographies were part of the standard treatment process. The inclusion criteria were as follows: (1) underwent coronary angiography with complete coronary angiography data; (2) aged 18 years and above and 44 years and below. The exclusion criteria were as follows: (1) those who had previously undergone coronary stent implantation or coronary artery bypass grafting, patients with tumors, inflammatory diseases, autoimmune diseases, moderate to severe renal insufficiency, cardiomyopathy, congenital heart disease, or rheumatic heart disease; (2) missing values in lipid indices; (3) missing values in covariates. Ultimately, 898 individuals were included in the analysis of this study. Figure 1 is the flowchart of the study.

### Data collection and definitions

Information was gathered from the hospital’s electronic medical record system. A standardized spreadsheet was designed to collect this retrospective information. Initial demographic and clinical information included age, sex, history of smoking, history of hypertension, history of diabetes, and family history of CHD. Laboratory measurements were made of fasting venous blood. Measurements were made of fasting blood glucose (FBG), HDL, LDL, TC, TG and Lipoprotein (a). The following equation was used to calculate the TyG index: In (fasting triglycerides (mg/dl) × fasting blood glucose (mg/dl)/2) [25].

### Delineation of lipid profiles

LPA was employed to delineate the lipid profiles. The Bayesian information criterion (BIC) is a commonly used fit metric. The number of classes was determined on the basis of the minimum BIC criterion. Additionally, a useful technique for class enumeration is the bootstrapped likelihood ratio test (BLRT) [26]. The *k*-class model is supported over *k*-1 classes by a statistically significant BLRT result. Theoretically, the model for which the BLRT first becomes statistically nonsignificant should be selected. The number of classes was determined using the BLRT. Finally, on the basis of the above blood lipids, the population was divided into four different lipid profiles.

### Outcome ascertainment

The results of CAG were interpreted using the Judkins method, and the Gensini score was independently assessed by two experienced cardiologists without access to the patients’ clinical information. In cases where the scores calculated by the two experts were inconsistent, a third physician was consulted to provide additional evaluation. Based on a Gensini score greater than zero, patients were categorized as either stenotic or nonstenotic. In accordance with accepted practices, patients were divided into mild to moderate and severe stenosis groups based on Gensini score tertiles in order to assess the severity of the condition [27–28]. Specifically, scores below 35 points indicated mild to moderate stenosis, whereas scores of 35 or higher were indicative of severe stenosis (severe CAS). When at least one major coronary artery, such as the left anterior descending, left main, left circumflex, and right coronary arteries, showed more than 50% luminal narrowing, CHD was diagnosed [29]. On the other hand, those with normal angiograms or less than 50% stenosis in any one coronary artery were categorized as non-CHD.

### Statistical analysis

For categorical variables, statistical descriptions in the form of frequencies and percentages were used, and the chi-square test was used to compare the differences in group composition. For continuous variables, the Shapiro‒Wilk test was applied to determine if they followed a normal distribution. Variables with a normal distribution were described using the mean ± SD, and differences between groups were analyzed via the two-independent samples t test. Conversely, nonnormally distributed variables were described by the median (with upper and lower quartiles), and the Wilcoxon rank-sum test was used for between-group comparisons. The restricted cubic spline method was used to explore the potential nonlinear associations between each lipid index and the risk of CHD with adjusting for confounders. Logistic regression models that incorporate additional demographic and clinical information were used to investigate the relationships between lipid profiles and CAS, severe CAS, and CHD. To avoid biases arising from sparse data issues, Firth’s penalized maximum likelihood estimation was employed to construct and estimate the logistic regression model. The predictive efficacy of models with lipid profiles alone and in combination with other clinical characteristics was evaluated by plotting receiver operating characteristic (ROC) curves and calculating the area under the curve (AUC), and the models were compared with models based on each single lipid index. Calibration curves were plotted for the prediction of coronary stenosis to assess the predictive performance of the models, and decision curves were plotted to assess the value of the model in clinical practice. All analyses were conducted using R 4.0.3 (R Foundation; available at http://www.R-project.org). The LPA model was estimated via the ‘mclust’ package in R. Statistical significance was determined at a two-tailed *P* < 0.05. The power analysis for the regression model was performed using G*Power (3.1.9.7) software.

## Results

### Main clinical features of the research population

This study involved 898 patients in all. The mean age was 38.03 years, and 814 (90.65%) males and 84 (9.35%) females were included. The mean Gensini score was 19.69. A total of 392 (43.65%) patients were diagnosed with CHD. LDL, HDL, TC, and TG levels were greater in the CHD group than in the non-CHD group, and FBG and TyG levels were higher as well. In addition, the distributions of Lp (a) and clinical characteristics (age, sex, history of smoking, history of hypertension, history of diabetes and family history of CHD) significantly differed between the CHD and non-CHD groups (Table 1).

**Table 1:** Demographic and laboratory characteristics stratified by CHD status

Characteristics	Total (*n* = 898)	Non-CHD (*n* = 506)	CHD (*n* = 392)	*P*
Age, years	39.00 (35.00–42.00)	38.00 (35.00–42.00)	40.00 (35.00–42.00)	0.043
LDL, mmol/L	2.67 (2.24–3.22)	2.50 (2.16-3.00)	2.89 (2.43–3.41)	< 0.001
HDL, mmol/L	1.01 (0.89–1.15)	1.04 (0.91–1.19)	0.98 (0.85–1.10)	< 0.001
TC, Umol/L	4.14 (3.56–4.77)	4.00 (3.44–4.55)	4.33 (3.70–5.02)	< 0.001
TG, mmol/L	1.68 (1.16–2.49)	1.52 (1.07–2.19)	1.90 (1.30–2.80)	< 0.001
FBG, mmol/L	5.05 (4.60–5.79)	4.89 (4.54–5.33)	5.41 (4.76–6.77)	< 0.001
TyG index	1.50 (1.04–1.97)	1.32 (0.93–1.78)	1.71 (1.28–2.14)	< 0.001
Lp (a), mg/dL	9.90 (5.40–20.20)	9.35 (4.80-19.15)	10.85 (6.00-21.53)	0.019
Sex, n (%)				< 0.001
Male	814 (90.65%)	443 (87.55%)	371 (94.64%)	
Female	84 (9.35%)	63 (12.45%)	21 (5.36%)	
History of smoking, n (%)				< 0.001
No	513 (57.13%)	328 (64.82%)	185 (47.19%)	
Yes	385 (42.87%)	178 (35.18%)	207 (52.81%)	
History of hypertension, n (%)				< 0.001
No	666 (74.16%)	401 (79.25%)	265 (67.60%)	
Yes	232 (25.84%)	105 (20.75%)	127 (32.40%)	
History of diabetes, n (%)				< 0.001
No	849 (94.54%)	492 (97.23%)	357 (91.07%)	
Yes	49 (5.46%)	14 (2.77%)	35 (8.93%)	
Family history of CHD, n (%)				
No	896 (99.78)	506 (100.00)	390 (99.49)	0.108
Yes	2 (0.22)	0 (0.00)	2 (0.51)	

LDL, low-density lipoprotein; HDL, high-density lipoprotein; TC, total cholesterol; TG, triglyceride; FPG, fasting plasma glucose; Lp (a), Lipoprotein (a); CHD, coronary artery disease

### Lipid profiles identified by LPA

**Fig. 2:**
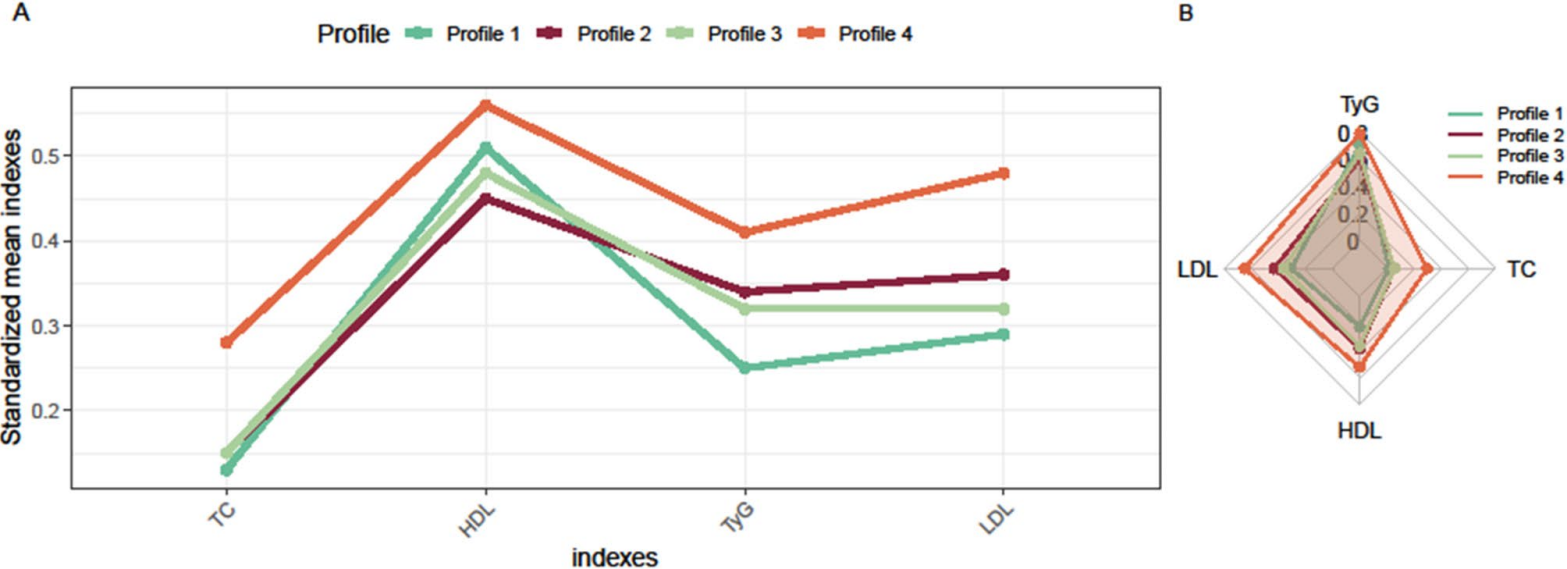
Lipid profiles identified by LPA. **A**, Line plot; (**B**) radar plot

Following model selection, four lipid profiles were identified as optimal. Figure 2 shows the standardized mean values of each lipid index for these four profiles, presented in both a line plot (A) and a radar plot (B). The profiles were categorized on the basis of the relative magnitudes of the lipid indices as follows: *Profile 1*, relatively high HDL with the lowest TC, LDL and TyG (41.20%). *Profile 2*: relatively high TC, LDL, and TyG with the lowest HDL (36.42%). *Profile 3*: relatively low TC, LDL, and TyG with relatively high HDL (18.93%). *Profile 4*: the highest TC, LDL, and TyG levels were associated with the highest HDL level (3.45%).

Gensini scores, coronary artery disease status, and the means and standard deviations of the lipid indices in the four profiles are displayed in Table 2. Notably, the distribution of each lipid index varied significantly across the profiles. Additional investigation into the relationship between each lipid index and the risk of CHD revealed a linear relationship between them, as shown in Fig. S1.

**Table 2:** Distribution of lipid indicators in the lipid profiles

Characteristic	*Profile 1* (*n* = 370)	*Profile 2* (*n* = 327)	*Profile 3* (*n* = 170)	*Profile 4* (*n* = 31)	*P*
TyG	1.22 ± 0.51	1.78 ± 0.65	1.72 ± 0.75	2.23 ± 1.33	< 0.001
LDL	2.52 ± 0.65	2.98 ± 0.79	2.76 ± 0.74	3.87 ± 1.91	< 0.001
HDL	1.08 ± 0.21	0.98 ± 0.17	1.02 ± 0.22	1.18 ± 0.41	< 0.001
TC	3.85 ± 0.74	4.36 ± 0.96	4.38 ± 1.02	7.37 ± 4.32	< 0.001
Gensini score	0.88 ± 1.07	47.72 ± 26.68	4.63 ± 2.74	31.18 ± 27.97	< 0.001
CAS					< 0.001
0	196 (52.97)	1 (0.31)	16 (9.41)	6 (19.35)	
1	174 (47.03)	326 (99.69)	154 (90.59)	25 (80.65)	
Severe CAS					
0	368 (99.46)	111 (33.94)	167 (98.24)	19 (61.29)	< 0.001
1	2 (0.54)	216 (66.06)	3 (1.76)	12 (38.71)	
CHD					< 0.001
0	357 (96.75)	12 (3.67)	127 (74.71)	9 (29.03)	
1	12 (3.25)	315 (96.33)	43 (25.29)	22 (70.97)	

### Associations between lipid profiles and ASCVD

Using *Profile 1* as the reference, logistic regression models were utilized to investigate the relationships between lipid profiles and the incidence of CAS, severe CAS, and CHD. Age, sex, the level of Lp (a), history of smoking, history of hypertension, history of diabetes, and family history of CHD were adjusted for as confounders in the model. With a given sample size of 898 and a significance level of 0.05, the power of the tests comparing the three categories of lipid profiles to the reference level reached above 80% for each outcome. Among the four lipid profiles, *Profile 2* had the highest risk of all types of ASCVD outcomes, whereas *Profile 1* had the lowest risk. With respect to CAS, *Profile 3* demonstrated a marginally elevated risk compared with *Profile 4*, whereas for severe CAS and CHD, *Profile 4* posed a greater risk than did *Profile 3* (Table 3). The results of the associations between the covariates and the occurrence of CAS, severe CAS, and CHD are shown in Table S1.

**Table 3:** Associations between lipid profiles and ASCVD

	β	OR (95%CI)	*P*
Lipids profiles with CAS			
*Profile 1*	Ref		
*Profile 2*	6.563	708.52 (102.099, 89461.392)	< 0.001
*Profile 3*	2.272	9.698 (5.721, 17.443)	< 0.001
*Profile 4*	1.494	4.456 (1.879, 12.101)	< 0.001
**Lipids profiles with severe CAS**			
*Profile 1*	Ref		
*Profile 2*	5.542	255.08 (87.579, 1233.757)	< 0.001
*Profile 3*	1.078	2.937 (0.566, 17.792)	0.194
*Profile 4*	4.41	82.3 (22.087, 449.548)	< 0.001
**Lipids profiles with CHD**			
*Profile 1*	Ref		
*Profile 2*	6.471	645.832 (302.487, 1522.401)	< 0.001
*Profile 3*	2.191	8.946 (4.735, 18.065)	< 0.001
*Profile 4*	4.185	65.7 (25.712, 182.78)	< 0.001

### Predictive performance of lipid profiles

**Fig. 3:**
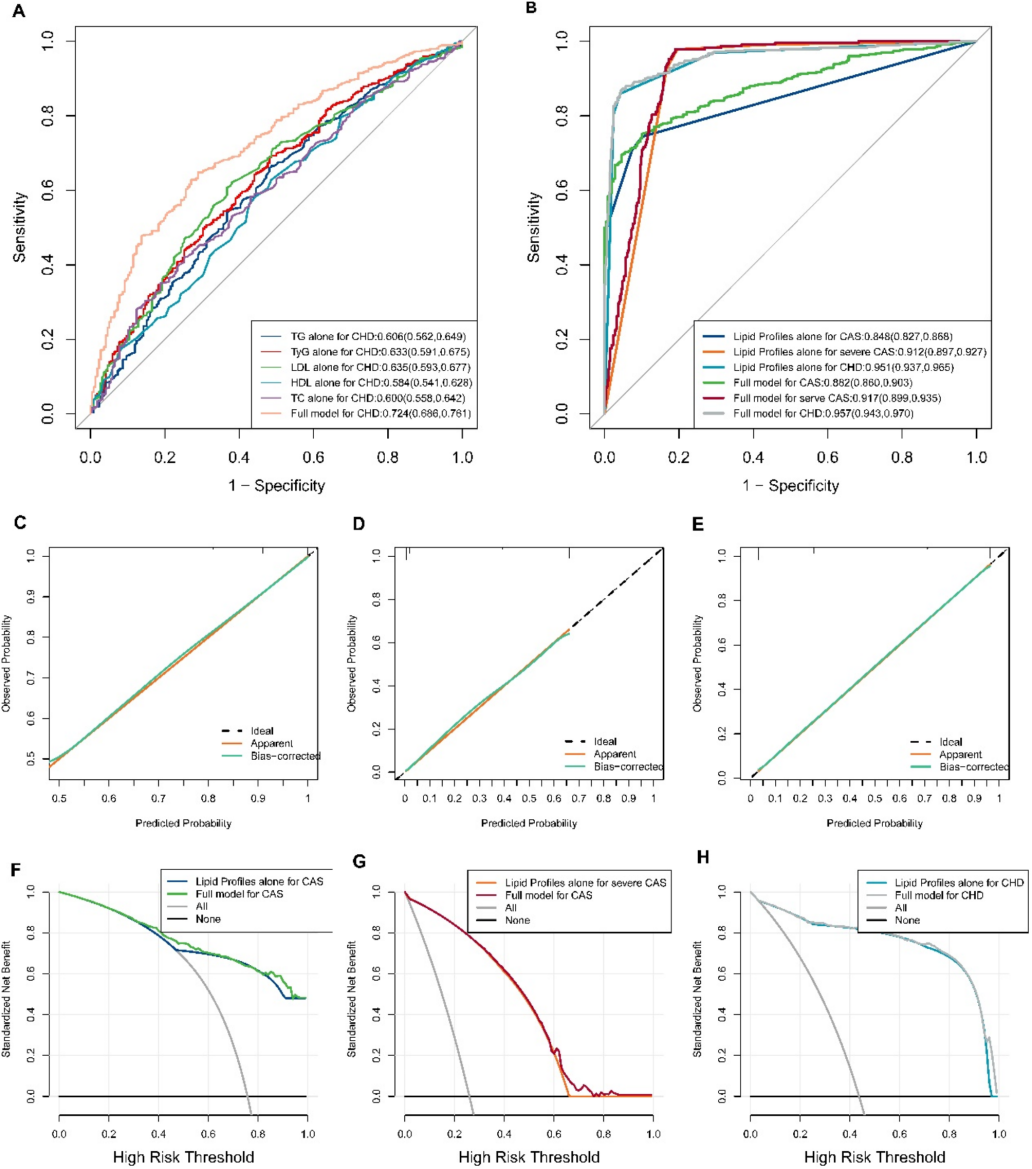
**A**, ROC curves of prediction models for CHD based on the lipid index alone and the linear combinations of the indices and other clinical characteristics. **B**, ROC curves for models predicting CAS, severe CAS and CHD on the basis of the lipid profile variable alone and the lipid profile in conjunction with other clinical characteristics. **C**-**E**, Calibration curves of models for CAS, severe CAS and CHD, respectively, on the basis of the lipid profile variable alone. **F**‒**H**, Decision curves of the model for CAS, severe CAS and CHD, respectively, on the basis of the lipid profile variable alone and the lipid profile in conjunction with other clinical characteristics

Figure 3 (A-B) displays the ROC curves for the models for predicting CAS, severe CAS, and CHD. These models were based on the lipid profile variable alone, the lipid profile variable in conjunction with other clinical characteristics, and the individual lipid index, as well as linear combinations of the indices. The AUC, sensitivity and specificity for each model are detailed in Table 4. Compared with models relying on individual lipid index or their combinations, the prediction model utilizing the lipid profile variable alone demonstrated significantly superior predictive accuracy. Furthermore, the inclusion of additional clinical characteristics alongside the lipid profile variable enhanced the predictive performance of the model, yielding excellent predictive accuracy for all outcomes. The calibration curves presented in Figure 3 (C-E) confirm the robust predictive performance of the model based solely on the lipid profile variable. Moreover, the decision curves in Figure 3 (F-H) underscore the significant potential of the model for clinical application.

**Table 4:** Predictive performance for CAS, severe CAS and CHD

For CAS	AUC	Sensitivity	Specificity
TyG	0.633 (0.591, 0.675)	0.690 (0.659, 0.720)	0.518 (0.486, 0.551)
LDL	0.635 (0.593, 0.677)	0.622 (0.590, 0.654)	0.619 (0.588, 0.651)
HDL	0.584 (0.541, 0.628)	0.628 (0.596, 0.660)	0.514 (0.481, 0.546)
TC	0.606 (0.562, 0.649)	0.666 (0.635, 0.697)	0.514 (0.481, 0.546)
Lp (a)	0.534 (0.490, 0.578)	0.613 (0.581, 0.645)	0.463 (0.431, 0.496)
Lipid Profiles alone	**0.877 (0.811**, **0.943)**	**0.744 (0.716**, **0.773)**	**0.899 (0.879**, **0.919)**
Full model	**0.882 (0.860**, **0.903)**	**0.703 (0.673**, **0.733)**	**0.950 (0.935**, **0.964)**
**For severe CAS**			
TyG	0.703 (0.665, 0.742)	0.715 (0.685, 0.744)	0.640 (0.609, 0.672)
LDL	0.667 (0.627, 0.708)	0.724 (0.694, 0.753)	0.563 (0.530, 0.595)
HDL	0.624 (0.584, 0.665)	0.768 (0.740, 0.795)	0.466 (0.433, 0.498)
TC	0.646 (0.605, 0.687)	0.649 (0.618, 0.680)	0.625 (0.594, 0.657)
Lp (a)	0.566 (0.524, 0.608)	0.691 (0.661, 0.721)	0.439 (0.407, 0.472)
Lipid Profiles alone	**0.912 (0.897**, **0.927)**	**1.000 (1.000**, **1.000)**	**0.806 (0.780**, **0.832)**
Full model	**0.917 (0.899**, **0.935)**	**0.979 (0.969**, **0.988)**	**0.811 (0.785**, **0.836)**
**For CHD**			
TyG	0.659 (0.623, 0.695)	0.607 (0. 575, 0.639)	0.672 (0.641, 0.703)
LDL	0.639 (0.602, 0.676)	0.635 (0.604, 0.667)	0.615 (0.583, 0.646)
HDL	0.595 (0.558, 0.632)	0.691 (0.661, 0.722)	0.482 (0.450, 0.515)
TC	0.608 (0.571, 0.645)	0.556 (0.524, 0.589)	0.642 (0.611, 0.674)
Lp (a)	0.546 (0.508, 0.584)	0.653 (0.622, 0.684)	0.439 (0.406, 0.471)
Lipid Profiles alone	**0.951 (0.937**, **0.965)**	**0.860 (0.837**, **0.882)**	**0.959 (0.945**, **0.972)**
Full model	**0.957 (0.943**, **0.970)**	**0.849 (0.826**, **0.873)**	**0.960 (0.948**, **0.973)**

## Discussion

This study innovatively identified four lipid profiles on the basis of four routine lipid indices with the help of a LPA model, demonstrating their strong associations with the occurrence of CAS, severe CAS, and CHD, as well as their excellent predictive diagnostic value.

Lipid indices have long been a central concern in coronary artery disease research. The traditional plasma lipid indices LDL, HDL, TG, and TC, as well as the TyG index, have been extensively studied. The linear correlations between the lipid indices and the risk of CHD demonstrated in this study are in line with current diagnosis and treatment guidelines [16] and are supported by numerous other studies [10, 16–17].

With the help of latent profile modeling, four distinct lipid profiles among patients were identified on the basis of four routine lipid indices (LDL, HDL, TG, and TyG). The characteristics of each profile could be described as follows: *Profile* 1 (relatively high HDL with the lowest TC, LDL and TyG): this group was defined by a relatively high level of HDL, which is protective, whereas the other three indices (TC, LDL and TyG) were the lowest across all profiles. *Profile* 2 (relatively high TC, LDL, TyG with the lowest HDL): this profile featured elevated levels of LDL, TG, and TyG above the normal range [10], although lower than those in *Profile* 4, while the HDL was relatively high but not the lowest across the profiles. *Profile* 3 (relatively low TC, LDL, TyG with relatively high HDL): this group was characterized by moderately low levels of TC, LDL, TyG compared with *Profile 2*, with HDL lower than both *Profile* 4 and *Profile* 1. *Profile* 4 (the highest TC, LDL, TyG with the highest HDL): this profile was notable for having the highest levels of all four indices, including the risk indices LDL, TG, and TyG, as well as the protective HDL. On the basis of the distinctive distribution of the four lipid indices, the profiles were named to reflect their predominant characteristics.

For the risk of cardiovascular diseases across the four lipid profiles, this study investigated the associations between the profiles and CAS, severe CAS, and CHD. The findings indicated that among the four profiles, *Profile* 1 posed the lowest risk. This finding aligns with the established notion that LDL, TC, and TyG are risk factors for cardiovascular outcomes [16–17, 30], whereas HDL plays a protective role [34]. Consequently, the combination of the lowest LDL, TC, TyG and high HDL was associated with the lowest risk of cardiovascular events. Furthermore, *Profile* 2, characterized by relatively lower levels of LDL, TC, and TyG and the lowest HDL, presented the highest risk for all adverse outcomes.

*Profile* 2, rather than *Profile* 4, had the highest risk for all adverse cardiovascular outcomes, which was somewhat unexpected given that *Profile* 4 had the highest levels of TC, LDL and TyG, which are well established as strong risk factors for cardiovascular diseases. These findings underscore the importance of a comprehensive lipid profile assessment in identifying individuals at elevated risk. For mixed hyperlipidemia, progress in the field of human genetics has pinpointed angiopoietin-like 3 (ANGPTL3) as a potential key for mitigating cardiovascular hazards. Genetic epidemiology shows that the functional absence of ANGPTL3 is strongly associated with reduced LDL levels, decreased triglyceride-enriched lipoproteins, and a lower risk of coronary artery disease [31–33]. The primary distinguishing risk factor for *Profile 2* over *Profile 4* was its lower HDL level. Thus, these findings emphasize the crucial role of HDL, a protective lipoprotein, in influencing cardiovascular outcomes.

Since the discovery of an inverse association between HDL and coronary artery disease in the 1950s, the cardioprotective benefits of high HDL levels have been extensively demonstrated [34]. The Framingham Heart Study in 1977 was instrumental in establishing HDL as a significant predictor of CHD, showing a stronger correlation with CHD incidence than LDL levels. Numerous subsequent studies have confirmed the epidemiological link between HDL levels and CHD risk [35–37], the severity of atherosclerosis [38], and mortality rates [39]. In addition, a meta-analysis that included 68 longitudinal prospective studies emphasized the crucial significance of measuring HDL levels in evaluating the risk of CHD [40].

While LDL remains a central focus for baseline lipid markers and is recommended as the main target for reducing lipids in the third edition of the NCEP guidelines [41], prior research has indicated that a substantial proportion of patients remain at elevated risk for coronary stenosis even after targeted LDL-lowering interventions [42–44]. Novel strategies and approaches are thus urgently required to further lower this risk. In this study, lipid *Profile 2*, which had a lower HDL level, despite having lower LDL, TC and TyG levels than did *Profile 4*, presented a greater risk for cardiovascular diseases because of its lower HDL levels. This highlighted the necessity of a thorough lipid profile evaluation in identifying individuals at elevated risk and suggested that HDL may warrant special consideration in the prevention and management of cardiovascular diseases.

Regarding the protective mechanism of HDL in atherosclerosis, the long-accepted theory is “reverse cholesterol transfer” (RCT), in which HDL helps move cholesterol from peripheral tissues to the liver for elimination [45]. In addition, HDL has been shown to suppress the expression of endothelial cell adhesion molecules, such as E-selectin, vascular cell adhesion molecule-1 (VCAM-1), and intercellular adhesion molecule-1 (ICAM-1) [46–45]. Additionally, HDL suppresses inflammation and the advancement of atherosclerosis by inhibiting the vascular smooth muscle cells’ and endothelial cells’ synthesis of monocyte chemotactic protein 1 (MCP-1) [47]. HDL also impedes atherogenesis by preventing LDL oxidation [48], inhibiting endothelial cell apoptosis [49], and modulating adherence of platelets by limiting the growth and activation of platelets [50].

Consistent with previous findings, this study underscores the significant role of HDL in cardiovascular disease development. The third edition of the NCEP guidelines places greater emphasis on HDL, raising the normal threshold [41]. This suggests that it may be possible to pay more attention to HDL in the prediction of the risk for coronary artery disease, as well as in treatment and management.

Therefore, this study suggests that integrating multiple lipid indices (TC, LDL, HDL, TG, and TyG) rather than a single index in clinical practice may enhance diagnostic and prognostic accuracy. For instance, individuals with high TC, LDL, and TyG but the lowest HDL had a higher cardiovascular event risk compared to other groups, particularly those with the highest LDL, TG, and TyG but also the highest HDL. This highlights the importance of a comprehensive lipid profile for patient diagnosis and risk assessment and underscores the potential risk associated with low HDL. Such patients may require closer monitoring and more vigilant care in clinical practice. This finding provides insights for personalized risk assessment and improved cardiovascular health strategies.

### Study strengths and limitations

This study has a number of noteworthy strengths. First, a LPA model was innovatively applied for the stratification of lipid indices and thus constructed novel lipid profiles, which could enhance the understanding of dyslipidemia subtypes. LPA classifies individuals by stratifying a series of variables, making it very suitable for delineating population lipid profiles. Second, a robust association was demonstrated between these lipid profiles and cardiovascular diseases, which is instrumental for identifying populations at elevated risk. Third, the predictive model confirmed the excellent ability of the new lipid profiles to predict cardiovascular outcomes, which validated the accuracy of the delineation of lipid profiles.

It is important to recognize the various limitations of the current study. First of all, the study population consisted of patients retrospectively enrolled from cardiology wards, most of whom were diagnosed with cardiovascular diseases after admission. This retrospective design means that the disease status may have altered the lipid profiles, making it challenging to establish clear causal relationships. Future prospective studies are needed to explore this relationship further. Additionally, this is a relatively young, single-center study with a significantly higher proportion of males than females among the participants, which may limit the generalizability of the lipid classification results to a broader population. To address this, future studies should consider expanding this analysis to include a more diverse and representative sample. Moreover, it may be interesting to compare the lipid profiles revealed in this young population with those in an older population. Furthermore, the sample size of this study was relatively small, which may limit the generalizability of the lipid profile classifications. Further investigation in larger populations is warranted to address this issue.

## Conclusions

Our study identified four distinct lipid profiles among the patients, revealing a nuanced landscape of cardiovascular risk. The profile characterized by lower TC, LDL and TyG levels, coupled with lower HDL, was associated with the highest risk, rather than the profile with the highest levels of these four lipid indices. These findings underscore the importance of comprehensive lipid analysis in detecting individuals at high risk. This insight could pave the way for more personalized risk assessments and improved clinical strategies for cardiovascular health.

## Electronic supplementary material

Below is the link to the electronic supplementary material.

Supplementary Material 1: Fig. S1 Relationships between TyG, HDL, LDL and TC with the risk of CHD by the restricted cubic spline (RCS) method. TC, total cholesterol; LDL, low-density lipoprotein; HDL, high-density lipoprotein; TyG, the triglyceride-glucose; CHD, coronary heart disease. Table S1 Associations between covariates and ASCVD.

## Data Availability

The research data supporting this study’s findings can be accessed through contact with the corresponding author, subject to appropriate justification and approval.

## Abbreviations

ASCVD: Atherosclerotic Cardiovascular Diseases
CAG: Coronary Angiography
TC: Total Tholesterol
LDL: Low-Density Lipoprotein
HDL: High-Density Lipoprotein
TG: Triglycerides
TyG: Triglyceride-glucose
LPA: Latent Profile Analysis
CAS: Coronary Stenosis
CHD: Coronary Heart Disease
MI: Myocardial Infarction
BIC: Bayesian Information Criterion
BLRT: Bootstrapped Likelihood Ratio Test
ROC: Receiver Operating Characteristic
AUC: Area Under the Curve
FBG: Fasting Blood Glucose
Lp (a): Lipoprotein (a)
ANGPTL3: Pinpointed Angiopoietin-like 3
RCT: Reverse Cholesterol Transfer
VCAM-1: Adhesion molecule-1
ICAM-1: Intercellular adhesion molecule-1
MCP-1: Monocyte chemotactic protein 1
