# Supplementary material for: Novel lipid profiles and atherosclerotic cardiovascular disease risk: insights from a latent profile analysis
Source: Lipids Health Dis. 2025 Feb 25;24:71. doi: 10.1186/s12944-025-02471-3 (PMC11854406; doi:10.1186/s12944-025-02471-3)
Supplement: Supplementary file 1 — Supplementary Material 1: Fig. S1 Relationships between TyG, HDL, LDL and TC with the risk of CHD by the restricted cubic spline (RCS) method. TC, total cholesterol; LDL, low-density lipoprotein; HDL, high-density lipoprotein; TyG, the triglyceride-glucose; CHD, coronary heart disease. Table S1 Associations between covariates and ASCVD. [file 12944_2025_2471_MOESM1_ESM.docx]

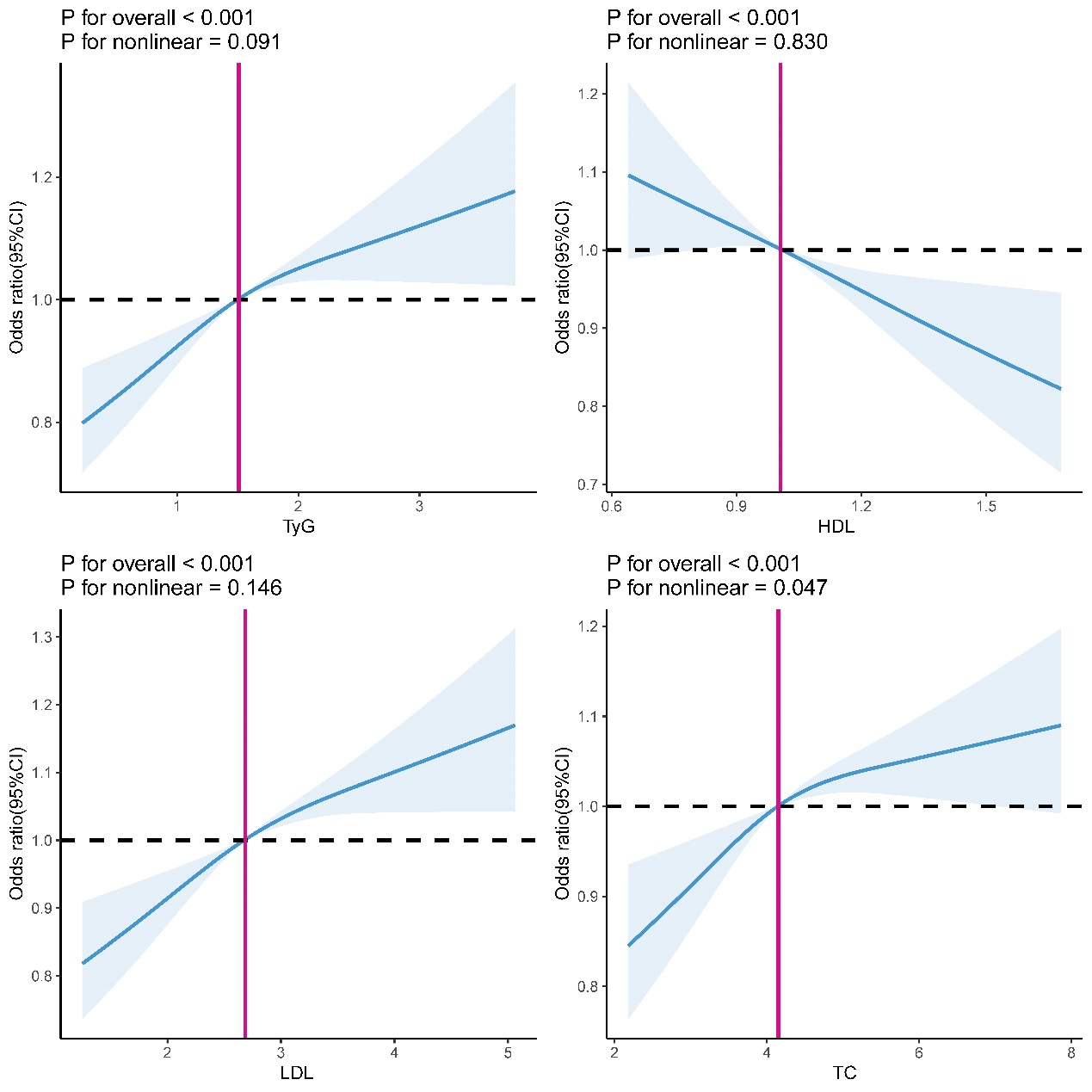


**SFig. 1** Relationships between TyG, HDL, LDL and TC with the risk of CHD by the restricted cubic spline (RCS) method. TC, total cholesterol; LDL, low-density lipoprotein; HDL, high-density lipoprotein; TyG, the triglyceride-glucose; CHD, coronary heart disease.

**Table S1 Associations between covariates and ASCVD**

|  | ***β*** | ***OR*(95%*CI*)** | ***P*** |
| --- | --- | --- | --- |
| **Lipids profiles with CAS** |  |  |  |
| Age (years) | 0.091 | 1.096(1.052,1.143) | <0.001 |
| Sex(Male vs. Female) | 0.340 | 1.404(0.775,2.553) | 0.262 |
| Lp(a) (≥30mg/dL vs. <30mg/dL) | 0.152 | 1.164(0.682,2.004) | 0.579 |
| History of Smoking(Yes vs. No) | 0.083 | 1.086(0.715,1.652) | 0.698 |
| History of hypertension (Yes vs. No) | 0.244 | 1.276(0.786,2.092) | 0.325 |
| History of diabetes (Yes vs. No) | -0.386 | 0.679(0.234,2.104) | 0.490 |
| Family history of CHD (Yes vs. No) | 0.180 | 1.198(0.051,185.06) | 0.915 |
| **Lipids profiles with severe CAS** |  |  |  |
| Age (years) | 0.000 | 1.000(0.956,1.046) | 0.989 |
| Sex(Male vs. Female) | 0.175 | 1.192(0.428,3.306) | 0.735 |
| Lp(a) (≥30mg/dL vs. <30mg/dL) | 0.507 | 1.661(0.920,3.097) | 0.093 |
| History of Smoking (Yes vs. No) | 0.106 | 1.112(0.711,1.735) | 0.642 |
| History of hypertension (Yes vs. No) | 0.279 | 1.321(0.834,2.111) | 0.236 |
| History of diabetes (Yes vs. No) | 0.030 | 1.030(0.483,2.296) | 0.940 |
| Family history of CHD (Yes vs. No) | 1.828 | 6.224(0.36,940.118) | 0.223 |
| **Lipids profiles with CHD** |  |  |  |
| Age (years) | 0.026 | 1.027(0.971,1.087) | 0.354 |
| Sex(Male vs. Female) | -0.09 | 0.914(0.391,2.251) | 0.840 |
| Lp(a) (≥30mg/dL vs. <30mg/dL) | 0.615 | 1.850(0.929,3.640) | 0.079 |
| History of Smoking (Yes vs. No) | 0.123 | 1.131(0.662,1.922) | 0.650 |
| History of hypertension (Yes vs. No) | 0.183 | 1.201(0.682,2.100) | 0.524 |
| History of diabetes (Yes vs. No) | 0.850 | 2.339(0.771,6.944) | 0.132 |
| Family history of CHD (Yes vs. No) | 0.580 | 1.785(0.074,275.481) | 0.725 |
